# Leveraging Multiomic Signatures to Predict Body Composition^☆^

**DOI:** 10.1016/j.advnut.2026.100626

**Published:** 2026-04-03

**Authors:** Sri Lakshmi S Devarakonda, David A Hughes, Christian Rodriguez, Marcus D Goncalves, Steven B Heymsfield

**Affiliations:** 1 Pennington Biomedical Research Center, LSU System, Baton Rouge, LA, United States; 2 Grossman School of Medicine, New York University, New York, NY, United States

**Keywords:** nutritional assessment, proteomics, metabolomics, adiposity, muscularity

## Abstract

Obesity, sarcopenia, malnutrition, and cachexia all include assessment of body composition in their respective clinical guidelines, although patient evaluations frequently require hardware that may not be available. Body mass index often fills this void, but its accuracy as a body composition phenotyping method is limited. Recent advances suggest a new option for assessing patient body composition: omics-derived circulating blood biomarkers applied in combination with demographic information or alone to estimate the mass or volume of clinically relevant body components. The origin of this movement has its roots, among other factors, in 2 longstanding observations: that serum concentrations of the metabolite creatinine are associated with total body skeletal muscle mass and that serum concentrations of the protein leptin are associated with total body adipose tissue mass. The first section of this review demonstrates how body composition prediction models can be developed that exploit these kinds of observations by adding serum metabolite and protein measurements to conventional equations designed to estimate components that include demographic covariates such as weight, height, sex, and body circumferences. The second section of this review then broadens the number of potential circulating blood metabolites and proteins examined by reviewing advances in omics technology that offer the potential to improve conventional body composition prediction models that include demographic data or to create models solely based on blood biomarker measurements. The final section of the review presents a perspective on this rapidly advancing area of human body composition assessment.

**Statement of Significance**

This perspective is the first to synthesize how blood biomarkers, notably those derived from multiomics, can be integrated with or replace demographic information to build prediction models for body composition assessment, a paradigm shift enabled by advances in omics and data analysis tools.

## Introduction

Evaluation of body composition is increasingly a focus of patient diagnosis, treatment, and monitoring [1,2]. Although multiple methods of quantifying body components are available [3,4], implementation of required hardware may not always be feasible in some clinical settings or circumstances. This technology void is often filled by BMI, a practical but limited body size and shape measure that cannot be used to accurately differentiate between major components such as adipose tissue and skeletal muscle. This well-recognized limitation of BMI could lead to systemic misclassification of body composition and metabolic risk in individuals whose body composition deviates from the assumptions underlying BMI and associated healthy weight cut points [5,6]. This reality brings us to the core of this commentary: exploring the potential of circulating blood biomarkers to estimate the mass or volume of body composition components. Blood samples are frequently available in clinical settings, and measured metabolites and proteins could be used in combination with demographic information or alone to estimate a person’s body composition, thus leading to improved diagnosis and clinical management. Although the focus of this review is on blood metabolites and proteins, urine is also a rich source of potential biomarkers. This is a narrative (perspective) review, designed to synthesize important concepts and highlight emerging directions rather than to deliver a protocol-driven evidence summary. To inform and contextualize our perspective, we conducted iterative searches in PubMed and on the open web (Google/Google Scholar), using topic-relevant key words and citation chaining from core publications.

Our review begins with a demonstration of how 3 blood biomarkers, serum creatinine, cystatin C, and leptin, can be used as covariates in skeletal muscle and total body adipose tissue prediction models that include available demographic variables such as weight, height, age, sex, and waist circumference (WC). A brief overview is provided for those unfamiliar with development of these types of conventional body composition prediction models. The section that follows casts a wider net examining the large and growing literature relating metabolomic and proteomic analyses to body components and their functions. High-throughput omics methods are advancing rapidly, and we therefore include a supplementary section (Supplementary Data I) that summarizes key relevant topics for those unfamiliar with this area. Our review concludes with an outlook for the potential of blood biomarkers to advance the science of human body composition assessment.

## Predicting Body Composition

### Conventional models

In this section, we demonstrate how individual and combinations of circulating blood biomarkers can be incorporated into conventional body composition prediction formulas that include readily available demographic covariates such as weight, height, age, sex, and WC. This approach serves as a framework for potentially expanding the conventional prediction models by increasing the number of circulating metabolites and proteins informed by omics evaluations as discussed later in this review.

Body composition prediction models, as described in this section, are usually developed based on multivariable linear regression. Explanatory variables in these models account for variation in an outcome of interest. Although some collinearity among explanatory variables is expected, each variable ideally contributes independently to the total explained variance. Prediction model performance is typically assessed through resampling methods such as *k*-fold cross-validation in which the dataset is divided into *k* equal parts. The model is trained on *k* − 1 folds and tested on the remaining 1-fold. This process is repeated *k* times so that each fold is used once as the test set. Commonly, 10-fold cross-validation is used. Across folds, model performance is evaluated using metrics such as the coefficient of determination (*R*^2^), root mean square error, and mean absolute error. If these statistics are similar across the *k* iterations of training and testing, then the model is considered to generalize well. Although WC has been incorporated, it is important to note that this metric is challenging to collect reliably in many clinical settings and is vulnerable to significant manual measurement error [7].

The final reported prediction model is then the result of re-estimating the body composition prediction equation on the entire data set which would then, ideally, be further validated using an independent external data set. This framework is also applied to more complex approaches like penalized regression and ensemble machine learning methods that are increasingly being used with omics datasets to predict complex traits such as body composition phenotypes.

### Skeletal muscle

#### Serum creatinine

Creatinine is a metabolite produced by the nonenzymatic irreversible cyclization of intracellular creatine phosphate, a high-energy intermediate distributed mainly in skeletal muscle and is secreted into the circulatory system at a relatively constant rate [8]. Creatinine, a metabolic end-product, is then excreted in urine by the kidneys. It has long been recognized that serum concentrations of creatinine are associated with muscularity, or the quantity of skeletal muscle [8]. In the absence of creatine ingestion, mainly in the form of dietary supplements [9], serum creatinine concentrations are correlated with total body creatine pool size and skeletal muscle mass in people with normal kidney function [[9], [10], [11]] (Supplementary Data II and Supplemental Figure S1 ). Consistent with creatinine’s stable generation rate in skeletal muscle and renal excretion, 24-h urine concentrations of creatinine are correlated with total body skeletal muscle mass (Supplemental Figures S2 and S3 [12]). Serum creatinine concentrations rise with onset of diseases impacting renal function and diminution of glomerular filtration rate (GFR). Thus, serum creatinine has the theoretical potential of being a good metabolite biomarker of skeletal muscle mass when dietary and clinical conditions are favorable including stable kidney function, consistency in protein intake without abrupt changes that alter creatinine production, and the absence of external influences such as creatine supplementation.

As a recent example of the link between serum creatinine and skeletal muscle, Yim et al. [13] observed that serum creatinine was associated with dual-energy X-ray absorptiometry (DXA)–measured appendicular (lean) muscle mass adjusted for height squared in the Korean NHANES: males (*n* = 456) and females (*n* = 1046); *r*^2^: 0.32 and 0.16, respectively, both *P* < 0.001. Appendicular lean mass is a surrogate marker of total body skeletal muscle mass [14].

Observations such as those of Yim et al. [13] and others [[15], [16], [17], [18]] (Table 1) related to creatinine metabolism can thus form the theoretical and empirical basis for developing conventional prediction equations for skeletal muscle mass that include this metabolite. To explore this hypothesis further, we examined the association between serum creatinine and total body skeletal muscle mass and developed prediction models that included anthropometric measurements and serum creatinine, using available data from the NHANES (Supplementary Data III). The evaluated sample characteristics are summarized in Table 2. Our analysis largely replicated the results of Yim et al. [13] (Table 3, model A, *R*^2^: 0.20), and the addition of creatinine to anthropometry-based models modestly improved model performance (model B→model C; *R*^2^: 0.89→0.90).

**TABLE 1:** Selected studies incorporating serum creatinine and cystatin C as predictors of skeletal muscle mass or function

Author (year)	Population	Age (y)	Phenotype predicted (method)	Biomarker type	Findings
Ballew et al. [15] (2023)	15,792 participants in the Atherosclerosis Risk in Communities Study	45–64	Frailty (identified with 5-component questionnaire)	CMI, a novel index based on SCr and SCysC	Lower CMI associated with higher frailty risk (OR: 4.23 males, 2.34 females).
Lin et al. [16] (2020)	272 patients with nondialysis chronic kidney disease	66.5 ± 15.1	SMI (by BIA)	SCr/SCysC ratio	SCr/SCysC positively correlated with SMI (*r*^2^ = 0.09) and handgrip strength (*r*^2^ = 0.12).
Bartholomae et al. [17] (2022)	82 healthy adults without obesity	19–50	LBM (DXA) Hand grip strength	SCr	SCr positively correlated with LBM (*r*^2^ = 0.42) and handgrip strength (*r*^2^ = 0.41).
Patel et al. [18] (2013)	118 hemodialysis patients	49 ± 12	LBM (DXA-derived)	SCr	Monthly predialysis SCr strongly and linearly correlated with LBM; regression equations using SCr accurately estimated LBM but underestimated at higher values.

Abbreviations: BIA, bioimpedance analysis; CMI, creatinine muscle index; DXA, dual-energy X-ray absorptiometry; LBM, lean body mass; OR, odds ratio; SCr, serum creatinine; SCysC, serum cystatin C; SMI, skeletal muscle index.

**TABLE 2:** Participant characteristics, NHANES data

Characteristic	Overall (*N* = 11,240)^1^	Male (*n* = 5723)^1^	Female (*n* = 5517)^1^
Survey year			
1999–2000	3431 (31%)	1757 (31%)	1674 (30%)
2001–2002	4023 (36%)	2045 (36%)	1978 (36%)
2003–2004	3786 (34%)	1921 (34%)	1865 (34%)
Age (y)	47 ± 20	46 ± 20	47 ± 20
Weight (kg)	78 ± 18	83 ± 16	73 ± 18
Height (cm)	168 ± 10	174 ± 8	161 ± 7
Waist circumference (cm)	95 ± 15	97 ± 14	93 ± 15
Skeletal muscle mass (kg)	25 ± 7	29 ± 5	20 ± 4
Creatinine (mg/dL)	0.88 ± 0.20	0.99 ± 0.17	0.78 ± 0.17
Cystatin C (mg/L)	0.75 ± 0.21	0.77 ± 0.19	0.74 ± 0.23

1 All continuous results are presented as mean ± SD, and categorical results are presented as *n* (%).

**TABLE 3:** Skeletal muscle prediction equations developed including serum creatinine and/or cystatin C as model covariates^1^

Model	Equation	*R*^2^ (RMSE)	*P* value
A	11.78 + 14.88 × SCr	0.20 (5.9)	<0.001
B	−10.8 − 5.05 × F − 0.05 × A + 0.15 × Ht + 0.21 × Wt	0.89 (2.16)	2
C	−11.41 − 4.49 × F − 0.06 × A + 0.13 × Ht + 0.21 × Wt + 3.49 × SCr	0.90 (2.09)	<0.001
D	25.27 − 0.45 × SCysC	0.0002 (6.62)	0.124
E	−10.07 − 4.29 × F − 0.05 × A + 0.12 × Ht + 0.21 × Wt + 5.58 × SCr − 3.67 × SCysC	0.91 (2.02)	<0.001
F	3.36 − 4.7 3 × F − 0.03 × A + 0.07 × Ht + 0.32 × Wt + 4.46 × SCr − 2.9 × SCysC − 0.14 × WC	0.92 (1.89)	<0.001

Abbreviations: A, age (y); F, female (1 = female, 0 = male); H, height (cm); RMSE, root-mean square error (kg); SCr, serum creatinine (mg/dL); SCysC, serum cystatin C (mg/ L); Wt, weight (kg); WC, waist circumference (cm).

1 Data are from NHANES years 2000–2001, 2002–2003, and 2003–2004 (*n*, 5723 males; 5517 females; total, 11,240). All models were evaluated using 10-fold cross-validation on the full dataset (no separate train/test split). The reported *P* values correspond to the significance of each variable when it is added to the model.

2 All covariates in model B contributed to the model performance (*P* < 0.001).

#### Serum cystatin C

Cystatin C, another potential candidate for skeletal muscle mass prediction, is a circulating protein product of all nucleated cells [19] that is often used to estimate GFR as a largely muscle-independent measure of renal function [20]. Serum cystatin C is orthogonal to serum creatinine; the former is associated closely with adiposity [21,22] and the latter with muscularity, as noted in the previous section. These differing but complimentary relations are synchronous with a growing literature reporting associations between combined indices of serum creatinine and cystatin C and skeletal muscle mass, or related clinical outcomes [18]. As one example, Lin et al. [16] demonstrated that the ratio of serum creatinine to cystatin C was associated with a measure of skeletal muscle mass (*R*^2^: 0.09; *P* < 0.001) and handgrip strength (*R*^2^: 0.12; *P* < 0.001). Additional examples of selected relevant studies are summarized in Table 1. Combining serum creatinine and cystatin C may thus improve the prediction of skeletal muscle mass. We tested this hypothesis using the NHANES sample described earlier and in Supplementary Data III. When cystatin C was added as a covariate along with serum creatinine to a conventional anthropometric model, it further increased prediction performance (Table 3, model C→model E; *R*^2^: 0.90→0.91). Adding an available anthropometric measurement, WC, further improved model E performance (model F; *R*^2^: 0.92). We additionally assessed models stratified by sex. These sex-specific models demonstrated reduced predictive performance relative to the pooled model. Full results of sex-stratified models are reported in Supplemental Table S1. Although the additional variance explained by including serum creatinine and cystatin C in these conventional body composition models is small, they could still hold clinical relevance. Even a marginal increase in the predictive accuracy can reduce misclassification of individuals with low muscle mass. In populations for whom data such as magnetic resonance imaging-measured skeletal muscle mass are unavailable, these modest increases in *R*^2^ could turn into meaningful improvement in precision at the individual level. Additionally, these observations serve as a precursor to the omics section to follow that describes the potential of adding vast numbers of circulating metabolites and proteins to prediction equations.

### Total body adipose tissue

#### Serum leptin

Serum leptin, a polypeptide hormone mainly secreted by white adipose tissue, is known to serve as a key indicator of body adiposity [23]. Gene expression of leptin is 2- to 3-fold higher in subcutaneous adipose tissue depots than in visceral depots, although secretion rates from both depots strongly correlate with circulating serum leptin concentrations (Pearson’s *r*: 0.84 and 0.73, respectively) [24]. Serum leptin concentrations in people with obesity are typically elevated due to increased total adipose-tissue mass and varying levels of leptin resistance [25]. Numerous studies have reported positive associations between serum leptin concentrations and body adiposity, although the magnitude of these associations is often modest [26,27]. In a controlled feeding study of postmenopausal females, percent body fat explained nearly 80% of the variance in serum leptin concentrations, outperforming BMI and other regional fat indices derived from DXA [28]. In a study involving a Japanese sample, Shimizu et al. [29] reported associations between serum leptin concentrations and total body fat mass (*r*^2^: 0.49; *P* < 0.0001), body fat percentage (*r*^2^: 0.31; *P* < 0.001), and BMI (*r*^2^: 0.40; *P* < 0.001).

These earlier findings, again both theoretical and empirical, indicate that serum leptin may serve as a predictor of overall adiposity when combined with available demographic information in estimation models. We tested this potential in a healthy adult sample (*n* = 99) evaluated in our laboratory (Supplementary Data IV and Supplemental Table S2) and found that leptin added significantly as a covariate to a conventional demographic-based fat mass prediction model (Table 4; model B→model C; *R*^2^: 0.77→0.81). This increase in explained variance reflects a better discrimination of the fat mass in an individual. Leptin provides a standardized, operator-independent biomarker that can reduce misclassification, especially when body size alone does not provide full insight. As with the serum creatinine-cystatin C model, WC further improved fat mass prediction (*R*^2^: 0.82). The relationship between fat mass and leptin, however, is context dependent. Measured leptin concentrations represent a single point in time and may not reflect variations that occur with changes in diet or other physiological conditions. In addition, increasing adiposity in individuals with overweight and obesity is often associated with leptin resistance, characterized by diminished central effects of leptin on appetite regulation [30]. Consequently, the linear relationship between serum leptin concentrations and adiposity may be attenuated or lost as adiposity increases.

**TABLE 4:** Total body adipose tissue prediction equations developed including serum leptin as a model covariate^1^

Model	Equation	*R*^2^ (RMSE)	*P* value
A	14.66 + 0.63 × Lep	0.36 (5.84)	<0.001
B	28.43 + 0.12 × age − 0.34 × Ht + 0.58 × Wt + 5.99 × F	0.77 (3.5)	2
C	17.60 + 0.10 × age − 0.24 × Ht + 0.49 × Wt + 3.72 × F + 0.31 × Lep	0.81 (3.19)	<0.001
D	−4.68 + 0.06 × age − 0.14 × Ht + 0.30 × Wt + 5.00 × F + 0.27 × Lep + 0.25 × WC	0.82 (3.06)	<0.001

Abbreviations: A, age (y); F, female (1, female; 0 male); Ht, height (cm); Lep, leptin (ng/mL); RMSE, root-mean square error (kg); Wt, weight (kg); WC, waist circumference (cm).

1 The data used to build these models were collected from a convenience sample of healthy adults taking part in studies conducted at the New York Obesity Nutrition Research Center’s Body Composition Unit, St Luke-Roosevelt Hospital, New York. Additional details are provided in Supplementary Data IV. Simple linear regression was performed, and model performance was assessed using *R*^2^ and RMSE calculated on the same dataset used for model fitting. N, 99.

2 All covariates in model B contributed to the model performance (*P* < 0.001).

As with serum creatinine and cystatin C, other adipose-derived hormones could potentially be combined with serum leptin to generate even stronger adipose tissue prediction models. From a clinical standpoint, creatinine is measured routinely, whereas cystatin C is increasingly recommended to confirm estimated GFR when creatinine is uncertain [31,32]. As one candidate, adiponectin represents the most abundant protein secreted by adipose tissue [33]. In contrast to leptin, plasma adiponectin is inversely related to BMI (*r*: −0.333, *P* < 0.0001) and adiposity (subcutaneous and intra-abdominal fat, *r*: −0.168, *P* < 0.05, and *r*: −0.35, *P* < 0.0001, respectively) [34]. Resistin and visfatin have also been previously identified as biomarkers of total body adiposity and its components, such as visceral adipose tissue [[35], [36], [37], [38]]. Measurements of these adipose-derived hormones are not routinely obtained in standard clinical care and are primarily used in research. Although no standard widely used commercial tests are available for most of these markers, some tests, such as adiponectin, are available. Similar tests could be developed for other markers in the future as evidence and clinical demand increase. Here again, we see the potential building blocks of body composition prediction models that can include multiple circulating proteins that form relatively stable relationships with total body and regional adiposity. That potential is strongly signaled by development in omics technology and research as reviewed in the next section.

### Omics methods and models

#### Background

**FIGURE 1:**
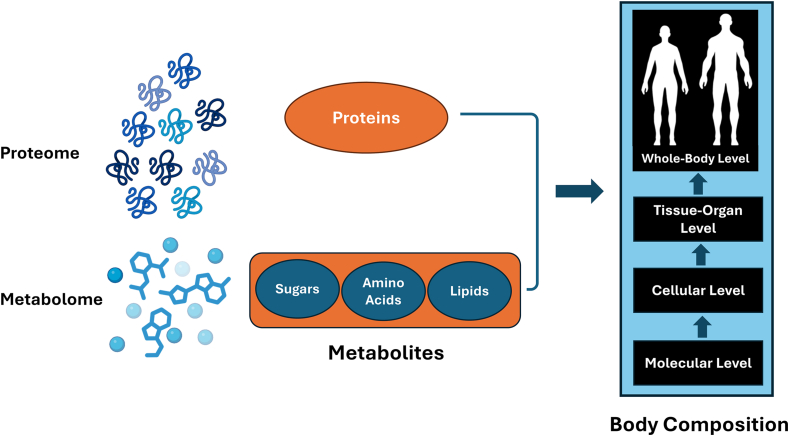
Association of the body’s major molecule classes with each other and body composition levels of organization. Molecules are grouped into the genome, transcriptome, proteome, and metabolome. Some circulating metabolites and protein biomarkers can form the basis of body composition prediction equations. *Created in part using BioRender.com.*

In contrast to standardized clinical chemistry assays that provide measurements of a few well-defined markers such as creatinine, cystatin C, or leptin, omics technologies enable the simultaneous quantification of hundreds to thousands of metabolites or proteins. This breadth allows for the biochemical complexity underlying diverse physiological outcomes, including body composition traits, to be captured more comprehensively. Metabolomics and proteomics are complementary approaches for profiling biological systems at the molecular level [39,40]. Although quantifying the abundance of every metabolite (the metabolome) and every protein (the proteome) in an organism would be ideal, current analytical platforms typically quantify only a few hundred or thousands of features. The term “feature” is typically used to describe a chemical compound’s analysis peak or signal. The concept advanced in the following discussion is that selected measurable blood proteins and metabolites, originating from their respective proteomes and metabolomes, maintain relatively stable associations with body components as depicted in Figure 1.

Metabolomics characterizes and quantifies small molecules (<1.5 kDa) that reflect metabolic pathway activity, genotype, disease states, and environmental influences such as diet, lifestyle, and behavior. Creatinine, already discussed as a muscle mass marker, is an example of a routinely detected metabolite in metabolomic studies.

Proteomics quantifies the abundance of proteins that serve as the functional products of gene expression and regulators of metabolic and structural processes. Like metabolites, protein abundance is also shaped by genotype, disease state, and the environment. Biomarkers such as cystatin C, leptin, and adipokines exemplify proteins commonly measured on proteomics platforms.

**FIGURE 2:**
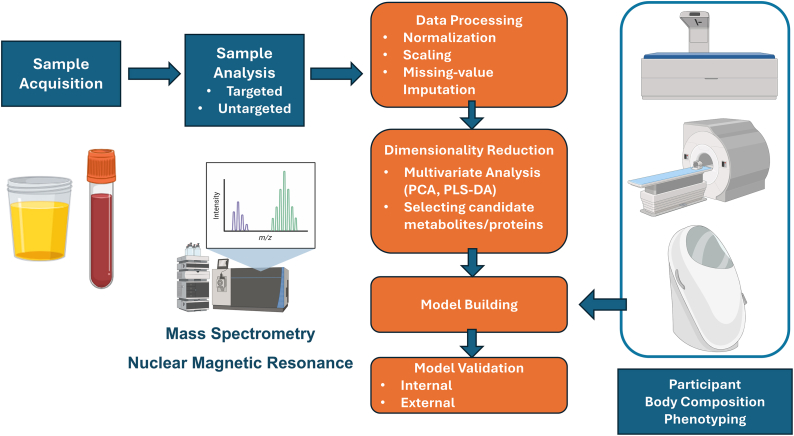
Schematic illustrating the workflow from biological sample collection to data-driven prediction modeling in omics research. Biofluids such as blood plasma or serum are collected, stored, and processed through extraction, chromatographic separation, and detection using technologies like mass spectrometry, nuclear magnetic resonance, or affinity-based assays. The resulting metabolomic or proteomic profiles undergo quality control and normalization, followed by optional dimensionality reduction using methods such as principal component analysis (PCA) and partial least squares discriminant analysis (PLS-DA). The processed omics data are then integrated with phenotypic body composition data obtained using established techniques such as dual-energy X-ray absorptiometry, magnetic resonance imaging, and BodPod (air displacement plethysmography). The resulting datasets are analyzed using machine learning approaches such as least absolute shrinkage and selection operator, ridge regression, elastic net, random forests, or gradient boosting to develop predictive models and identify key predictors or omics signatures. Created in part using BioRender.com.

Together, metabolomics and proteomics provide broad, complementary snapshots of an individual’s biochemical and physiological state. Such multidimensional molecular profiles can be leveraged to predict a wide range of complex traits, including body composition [41,42]. Figure 2 illustrates the workflow for constructing a prediction model using these multidimensional metabolomic and proteomic profiles, highlighting key steps from data acquisition to model development and validation.

The existing body of research that applies metabolomics and proteomics to body composition can be broadly categorized into 3 thematic areas. First, association studies, which identify metabolites or proteins associated with body composition traits that provide biological insights into relevant functional pathways [[43], [44], [45], [46], [47], [48], [49], [50], [51]]. Second, classification studies, which use omics profiles to distinguish individuals based on categorical traits such as high compared with low body fat or muscle mass [[52], [53], [54], [55]]. Third, quantitative prediction studies, which aim to estimate continuous body composition measures, such as total fat, lean mass, or organ-specific adiposity, using molecular data alone or in combination with demographic covariates [42,54,[56], [57], [58]]. The latter group, quantitative prediction models, forms the focus of this section. Selected studies integrating multiomics measurements as predictors of body composition are summarized in Table 5 in chronological order and are presented here in additional detail.

**TABLE 5:** Selected previous studies incorporating multiomic measurements as predictors of body composition

Author (y) (*N*)	Outcome	Omics type	Platform	Prediction method	Starting features (*n*)	Features included (*n*)	Key omics signatures/top-ranked features	*R*^2^ train (test)
Lim et al. [56] (2012) (*N* = 60)	Total FM	Proteo + Metabol + Lipids	Various	Random forest	48	5	Leptin, leptin-adiponectin ratio, free estradiol, plasminogen activator inhibitor-1, alanine transaminase	NR (0.69)
Lim et al. [56] (2012) (*N* = 60)	Trunk-periphery fat ratio	Proteo + Metabol + Lipids	Various	Random forest	48	5	Soluble leptin receptor, 25(OH)-vitamin D3, insulin-like growth factor binding protein-1, Coenzyme Q10	NR (0.35)
Lim et al. [56] (2012) (*N* = 60)	Visceral fat area	Proteo + Metabol + Lipids	Various	Random forest	48	5	Leptin, leptin-adiponectin ratio, C-reactive protein, lycopene, vitamin D3	NR (0.58)
Williams et al. [42] (2019) (*N* = 11,472)	% Body fat	Proteo	SomaLogic	Elastic net	5000	219	Top 3 features: Leptin, FABP4, SFRP4	0.92 (0.92)
Williams et al. [42] (2019) (*N* = 11,472)	LBM	Proteo	SomaLogic	Elastic net	5000	115	Top 3 features: SEZ6L, SLIK4, WISP-2	0.83 (0.82)
Williams et al. [42] (2019) (*N* = 11,472)	Visceral fat	Proteo	SomaLogic	Elastic net	5000	96	Top 3 features: leptin, FABPA, INHBC	0.71 (0.7)
Wu et al. [58] (2021) (*N* = 66)	VAT/SAT ratio	Metabol	LC/MS	PLS (MUVR)	910	31	Triglyceride-related markers, lipid features	0.7 (0.62)
Titova et al. [57] (2023) (*N* = 4950)	FMI	Proteo	Olink	Random forest (MUVR-RF)	261	105	Leptin, FABP4, IL-1ra, other immunity, inflammatory signaling markers, markers related to growth and angiogenesis.	0.72 (0.73)
Pathmasiri et al. [54] (2024) (*N* = 104)	% Body fat	Metabol	LC/MS	LASSO	10,535	NR	NR	0.64 (NR)
Pathmasiri et al. [54] (2024) (*N* = 104)	Sagittal abdominal diameter	Metabol	LC/MS	LASSO	10,535	NR	NR	0.67 (NR)

*Abbreviations:* CV, cross validation; FM, fat mass; FMI, fat mass index; FABP4, fatty acid–binding protein 4; GDF-15, growth differentiation factor 15; L-1ra, IL-1 receptor antagonist; INHBC, inhibin subunit beta C; LASSO, least absolute shrinkage and selection operator; LBM, lean body mass; NR, not reported; Metabol, metabolomics; LC/MS, liquid chromatography-mass spectroscopy; OOB, out-of-bag; PLS (MUVR), partial least squares (multivariate unbiased variable reduction); Proteo, proteomics; SAT, subcutaneous adipose tissue; SFRP-4, secreted frizzled-related protein 4; SEZ6L, seizure-related 6 homolog-like; SHBG, sex hormone–binding globulin; SLIK4, SLIT and neurotrophic receptor tyrosine kinase-like protein 4; VAT, visceral adipose tissue; WISP-2, WNT1-inducible signaling pathway protein 2.

#### Machine learning

Omics modeling takes a data-driven or hypothesis-free approach, with hundreds or thousands of explanatory variables; conventional linear regression approaches, as described earlier, become limited by overfitting and collinearity among predictors. To address this, penalized regression methods such as least absolute shrinkage and selection operator (LASSO), ridge regression, and elastic net are commonly applied. These methods extend linear regression by adding penalties that constrain or shrink regression coefficients. LASSO sets many coefficients to zero, effectively performing variable selection. In contrast, ridge regression shrinks coefficients toward zero without eliminating predictors. This improves model stability when features are highly correlated. Elastic net models combine both LASSO and ridge regression penalization methods. As such, elastic net models simultaneously perform variable selection and coefficient shrinkage. In all cases, *k*-fold cross-validation is used to determine the optimal penalization parameter that maximizes predictive performance.

When nonlinear relationships are expected between predictors and outcomes, ensemble machine learning methods such as random forests and gradient boosting may be better suited. These algorithms build large collections, or ensembles, of decision trees and combine their predictions to improve model accuracy. Random forests average predictions across many trees to reduce variance, whereas gradient boosting sequentially builds trees that correct the errors of prior iterations, enhancing accuracy but requiring more careful tuning. Ensemble methods can be more flexible and powerful but also more complex, with a greater number of parameters to optimize.

These approaches provide powerful tools for integrating metabolomic and proteomic data with demographic and clinical data. They can accommodate the large number of intercorrelated molecular features typical of omics data, improving prediction and accuracy while also revealing biologically relevant markers that may be missed by conventional linear models.

#### Body composition models

A prominent early example of the omics approach is the work of Lim et al. [56] who evaluated 60 postmenopausal females and showed that combining anthropometric measures with a starting set of 48 metabolic biomarkers, including adipokines, sex hormones, inflammatory proteins, and micronutrients, improved prediction of DXA-derived total fat mass, trunk-to-periphery fat ratio, and visceral fat area. Using random forest modeling without feature selection, their omics-only models, in which they included just the top 5 molecular predictors, achieved *R*^2^ values of 0.69, 0.35, and 0.58, respectively. In each instance, these models explained less variance than clinical models that included sex, ethnicity, and body size measures. However, when omics and anthropometric data were combined, *R*^2^ values increased to 0.91, 0.58, and 0.68, respectively. This pattern of increasing predictive value of combined blood marker and demographic information is similar to examples we presented earlier for skeletal muscle and adipose tissue.

In what is perhaps the most comprehensive study to date, Williams et al. [42] analyzed data from the Fenland Study (*n* = 11,472) that included 5000 proteins measured on SomaLogic’s affinity platform (SomaLogic Inc.). The authors developed models for percent body fat, lean body mass (LBM), and visceral fat. Using elastic net modeling, the group identified models that included 219, 115, and 96 proteins that explained 92%, 82%, and 70% of the variance in the validation dataset, respectively. Importantly, the proteomics-only models for percent body fat and LBM outperformed the best clinical models, both of which had *R*^2^ values of 0.75. Their best clinical models typically incorporated demographic information such as age, sex, and anthropometrics (height and weight). Leptin, fatty acid–binding protein (FABP), and secreted frizzled–related protein 4 were the top 3 features predicting percent body fat. Leptin, FABP A, and inhibin subunit beta C were the top 3 features for their visceral fat model. For LBM prediction, seizure-related 6 homolog-like, SLIT and neurotrophic receptor tyrosine kinase-like protein 4, and WNT1-inducible signaling pathway protein 2 were the top 3 features. Although some of these features that emerged in the LBM model did not show direct biological relevance to the target tissue, broader signatures showed multiple skeletal muscle contractile proteins. Comparison of the visceral fat models from the studies by Williams et al. [42] and Lim et al. [56] indicates that larger proteomic feature sets and use of an algorithm allowing for the inclusion of more independent predictors increased explanatory power by ∼12%. Notably, leptin emerged as the top biomarker in the models of both studies. FABP found in adipocytes was the second strongest biomarker for percent body fat and visceral fat models.

Wu et al. [58] analyzed 68 healthy females using 910 untargeted metabolomic features and identified a subset of 31 metabolites that together predicted the visceral-to-subcutaneous adipose tissue ratio. The metabolomic signature identified in this study was dominated by triglyceride species and acylcarnitines. The model in the training data set explained 70% of the variance, and in the validation set it explained 63% of the variance (*R*^2^: 0.70 training; *R*^2^: 0.63 validation).

Titova et al. [57] analyzed a cohort of 4950 females and measured 261 proteins using Olink’s (Olink Bioscience, Uppsala, Sweden) affinity technology to build a prediction model for DXA-derived fat mass index (FMI, kilogram per meter squared). Using random forest modeling along with variable selection using multivariate methods with unbiased variable selection, the authors built a 105-feature model that explained 73% of the variance in the validation set (*R*^2^: 0.72 training; *R*^2^: 0.73 validation). Again, the proteins with the strongest association with the outcome, FMI, were leptin and FABP 4.

Pathmasiri et al. [54] used 2 groups of 52 individuals (*n* = 104) that differed markedly in their body composition traits, along with 10,535 mass spectrometry-derived metabolite features to predict percent body fat and sagittal abdominal diameter. Using LASSO modeling with leave-one-out cross-validation, the authors reported *R*^2^ values of 0.64 and 0.67, respectively. The authors did not report how many features had a nonzero weight in the final models. However, metabolite features that classified the study population into 2 groups that varied in body composition were enriched in bile acid biosynthesis, histidine metabolism, and lysine metabolism pathways, among others.

As observed in the studies above, leptin and FABPs are frequent predictors of adiposity-related traits, consistent with their biology as adipocyte-derived proteins. However, as noted earlier in this perspective, some analytes exhibit nonlinear relationships with body composition traits. For example, circulating leptin concentrations may plateau or change at different rates at higher levels of BMI. In such circumstances, prediction models that rely on linear assumptions may underperform or yield variability in their accuracy across the distribution of the outcome. Consequently, ensuring that training and test modeling approaches capable of capturing nonlinear relationships may be necessary to build reliable and generalizable prediction models.

Collectively, these studies and those summarized in TABLE 1, TABLE 4 demonstrate the feasibility and growing sophistication of predicting body composition traits from molecular data. Model performance has steadily improved with larger sample sizes, broader omics coverage, and algorithms capable of handling high-dimensional data. Nevertheless, variation in study design, analytical platforms, and validation strategies complicates direct comparison across studies, a point further considered in the following perspective section.

## Perspective and Conclusions

Advances in high-throughput omics now create the opportunity for body composition to be modeled from circulating molecular profiles, yet their practical and biological advantages over traditional component analysis approaches remain to be fully defined. Previous work, using classical anthropometric features, such as weight, height, age, and WC, has already achieved high predictive accuracy for fat and lean mass (*R*^2^: 0.85–0.93) [59]. These observations are supported by the conventional body composition models developed in the current study (*R*^2^: 0.77–0.89, TABLE 2, TABLE 3). This raises the question, what additional value can metabolomics and proteomics bring to this field?

In many instances, omics data should be viewed as complementary to other measurements. Combining molecular features with traditional demographic covariates improves model precision, as shown by the examples for serum creatinine, cystatin C, and leptin. Omics-only models, in contrast, can offer mechanistic insight into the traits being modeled, particularly when the included features or their pathways are interrogated in detail. Moreover, residuals from predicted compared with observed values may themselves be informative for disease risk. For example, differences between proteomic-predicted and observed age have been associated with multiple health outcomes, including all-cause mortality [60]. Similarly, the difference between predicted and observed skeletal muscle mass could help identify individuals at higher risk or faster rates of sarcopenia or cachexia development.

Molecular prediction of body composition traits should also be evaluated within a sex-specific framework. Both body composition and molecular profiles differ markedly between males and females, and within-sex validation is necessary to properly assess model accuracy [61]. This does not mean that sex-specific models are always required, but performance metrics should be reported both in the combined sample and within each sex. If substantial differences are observed, then sex-specific modeling may be justified. For example, creatinine varies strongly by sex and performs poorly as a predictor when assessed by sex.

It is encouraging, however, that omics data alone can predict demographic and anthropometric traits such as sex, weight, height, and age [62]. This implies that molecular profiles encode systemic physiological information, enabling prediction of complex traits even in the absence of demographic data. Clinically, this concept aligns with the emerging idea of a “liquid health check” or a snapshot of one’s molecular physiology that can be used to infer overall health [42]. It also suggests the possibility of constructing a physical “avatar” of an individual using predicted demographic, anthropometric, and body composition traits [63,64].

Although prediction algorithms cannot imply causality by themselves, evidence from randomized controlled trials and Mendelian randomization studies indicates that body composition traits causally influence interindividual variation in metabolites and proteins [44,45,50,[65], [66], [67], [68], [69], [70], [71], [72]]. Such findings enhance the biological relevance of predictive models and suggest that their constituent features may, in part, represent causal mediators or biomarkers of metabolic health and disease risk.

Metabolomic and proteomic models also have growing translational potential for clinical risk prediction [41,42,73,74]. As omics profiling becomes more affordable, longitudinal monitoring of molecular signatures could enable yearly risk assessments for disease traits such as the cardiometabolic outcomes of type 2 diabetes. Currently, these applications are largely confined to research, for example, evaluating cardiometabolic fitness following randomized controlled trials [75]. As models for body composition traits become more refined, their potential clinical utility should also be considered, specifically how they might improve health assessment, treatment targeting, or monitoring of intervention response.

Harmonization of data across platforms remains one of the largest technical hurdles for this field. The 2 major commercial proteomics platforms, Olink and SomaLogic, show only modest concordance, with median cross-platform Spearman correlations of 0.33–0.53 [76,77]. This lack of agreement illustrates the challenge of reproducibility, which is further compounded by variation across university-based nuclear magnetic resonance or mass spectrometry cores. Achieving reproducible reference models will require common, standardized platforms. In practice, this likely means relying on a limited number of widely accessible reference technologies, such as those offered by Olink, SomaLogic, Nightingale Health (Nightingale Health Plc.), Metabolon, General Metabolomics, or core facilities like the Broad Institute. Doing so would ensure consistent and replicable translational modeling. Ideally, a large, geographically diverse population measured across multiple platforms could serve as a common reference for developing and validating such models.

Omics-based prediction of body composition offers considerable promise. It is not intended to replace established methods but rather to extend them. Metabolomic and proteomic data coupled with predictive modeling can deepen our mechanistic understanding of human physiology, enable integrative modeling across traits, and support clinical translation. To realize this potential, open and transparent data sharing, accessible model code, and systematic cross-cohort validation will be essential to ensure reproducibility and accelerate progress toward clinical implementation [78,79].

## Author contributions

The authors’ responsibilities were as follows – SBH, SLSD: conceptualized the perspective, conducted the literature review, and drafted the initial manuscript; DAH, SLSD, SBH: performed data analysis and carried out major revisions to the draft; MDG, CR: contributed to the refinement of the final draft; and all authors: read and approved the final manuscript.

## Data availability

Data described in the manuscript are available online at the NHANES website (https://www.cdc.gov/nchs/nhanes/index.html), from referenced studies, or, in the case of convenience samples, described in Supplementary Data, by request from the authors. Analytical code has been made publicly available on GitHub (https://github.com/SriDevarakonda1/bc_pred).

## Declaration of Generative AI and AI-assisted technologies in the writing process

During the preparation of this work, limited use of Microsoft Copilot (Microsoft 365), powered by the GPT‑5 chat model, was made solely for sentence‑level review of grammar and clarity. All substantive writing, interpretation, and final wording were performed by the authors, who reviewed and edited the manuscript in full and take complete responsibility for its content.

## Funding

This work was partially supported by National Institutes of Health NORC Center Grants P30DK072476, Pennington/Louisiana, P30DK040561, Harvard, R01DK109008, CGCATF-2021/100022, and OT2CA278685.

## Conflict of interest

SBH serves on the Medical Advisory Boards of Tanita Corporation, Novo Nordisk, Lilly, Abbott, Regeneron, and Medifast. MDG holds equity in Faeth Therapeutics and Skye Biosciences; reports consulting or advisory roles with Almac Discovery, Genentech Inc., Faeth Therapeutics, Scorpion Therapeutics, and Skye Biosciences; and patents, royalties, and other intellectual property with Weill Cornell Medicine and Faeth Therapeutics. All other authors report no conflicts of interest.
