# Supplementary material for: Leveraging Multiomic Signatures to Predict Body Composition
Source: Adv Nutr. 2026 Apr 3;17(5):100626. doi: 10.1016/j.advnut.2026.100626 (PMC13146523; doi:10.1016/j.advnut.2026.100626)
Supplement: multimedia component 1 [file mmc1.docx]

**SUPPLEMENTARY DATA**

**Leveraging Multi-omic Signatures to Predict Body Composition**

Sri Lakshmi S. Devarakonda^1^, David A. Hughes^1^, Christian Rodriguez^1^, Marcus D. Goncalves^2^, Steven B. Heymsfield^1^

^1^Pennington Biomedical Research Center, LSU System, Baton Rouge, LA, USA; ^2^[Department of Medicine, NYU Grossman School of Medicine, NY, USA](https://med.nyu.edu/medicine%22%20%5Ct%20%22_blank)

**Address Correspondence to:**

Sri Lakshmi S. Devarakonda, PhD, RDN

Pennington Biomedical Research Center

6400 Perkins Road

Baton Rouge, LA 70808

Tel : 225-763- 0161

Fax : 225-763-3030

E-mail: Sri.Devarakonda@pbrc.edu 

**Index**

**I. Omics Measurement Methods
II. Association of Serum Creatinine with Creatine Pool Size
III. NHANES Prediction Model Methods and Sex Specific Equations**

**IV. Pennington Biomedical Prediction Model Methods
Supplemental References**

**Supplementary Data I. Omics Measurement Methods**

Where omics technology begins to differ from standardized clinical assays is in its scale and analytical complexity. Mass spectrometry, combined with liquid and gas chromatography to reduce sample complexity, is a common technology used in metabolomics and proteomics. Chromatography separates individual molecules before detection, thereby improving detection and quantification accuracy. Other technologies, such as proton nuclear magnetic resonance (^1^H-NMR) and high-throughput affinity-based assay, are conceptually similar to enzyme-linked immunosorbent assays (ELISAs; e.g., that measure specific blood proteins) and are also commonly used in omics evaluations. For example, Nightingale Health’s (Helsinki, Finland) ^1^H-NMR platform focuses largely on lipoprotein subclasses while also quantifying selected metabolites, highlighting how commercially available NMR-based metabolomics emphasizes lipid metabolism.

Mass spectrometry-based assays, such as those deployed by Metabolon (Morrisville, NC, USA) or General Metabolomics (Cambridge, MA, USA), can be applied in targeted or untargeted modes. Target approaches focus on predefined molecules, often with internal standards that enable absolute quantification. Untargeted approaches aim to profile as many molecular features as possible, typically providing semi-quantitative or relative abundance data that may include unannotated or unidentifiable features. Affinity-based proteomic assays, such as those developed by Olink (Uppsala, Sweden) and SomaLogic (Boulder, CO, USA), similarly generate semi-quantitative data across thousands of proteins, while ^1^H-NMR provides absolute concentrations but generally for a smaller set of abundant metabolites and lipoproteins.

Obtaining metabolomic or proteomics data is, in many ways, analogous to collecting standard clinical chemistry data. The process begins with selecting the biological sample of interest and then running through the procedure of sample collection, storage, extraction, feature identification, quantification, normalization, and ultimately statistical analysis.

While solid tissue biopsies can be analyzed, biofluids are preferred because they are less invasive and easier to collect. Among possible options such as sweat, saliva, tears, breast milk, or urine, blood plasma is the most common and preferred sample type. Blood serum is also frequently used, though clotting processes can release platelet proteins and alter metabolite levels. The choice of tissue or biofluid should always reflect the underlying research question, but circulating venous blood is often a practical and physiologically relevant source as it transports molecules throughout the body.

It is important to note however that changes observed in the abundance of metabolites or proteins in circulation may not mirror those occurring within a target tissue of interest such as skeletal muscle. For example, during periods of muscle atrophy protein levels may decline within skeletal muscle while increasing in circulating venous blood, potentially leading to misleading inferences if sample context is not considered.

In practice, the complex steps following collection and storage are handled by specialized core facilities that return large data matrixes of metabolite or protein abundances, accompanied by quality control metrics, batch information, and feature annotations. Once quality-controlled and normalized these data can then be integrated with other phenotypic data to identify associations or predict complex traits, including body composition phenotypes.

**Supplementary Data II. Association of Serum Creatinine with Creatine Pool Size**

The association of serum creatinine with creatine pool size is shown in **Supplemental Figure S1** for a sample of 49 male participants in the study reported by Pasiakos et al. (1). Evaluations were at baseline with creatine pool size evaluated with D_3_-creatine dilution (2). 


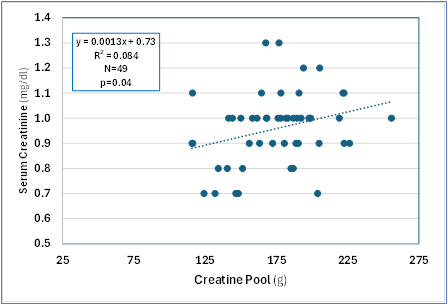


**Supplemental Figure S1.** Serum creatinine versus creatine pool size.

**Association of skeletal muscle mass in adults with 24-hour urine creatinine.** The data collected in **Supplemental Figure S2** was from a larger study reported by Pierson et al. (3). Skeletal muscle mass was measured with total-body magnetic resonance imaging, and three serial urine collections were analyzed for creatinine. Participants were 19 adults, 4 males and 15 females. Another example is shown in **Supplemental Figure S3** that included 8 males as reported by Wang et al. (4).


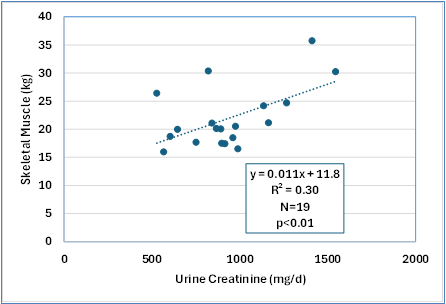


**Supplemental Figure S2**. Skeletal muscle mass versus 24-hour urine creatinine.


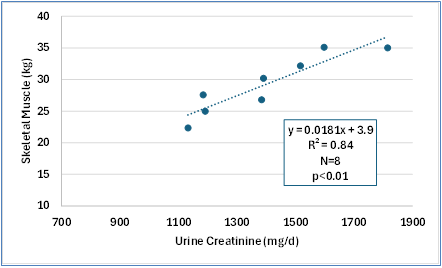


**Supplemental Figure S3**. Skeletal muscle mass versus 24-hour urine creatinine. 

**Supplementary Data III. Serum Creatinine and Cystatin C Prediction Model Methods**

The sample included three NHANES cycles, 1999-2000, 2001-2002, and 2003-2004. The total sample included 42,637 participants across all evaluated race/ethnic groups. Of those, 14,012 participants were excluded who were < age 18 years and 8841 participants were removed due to having serum creatinine >1.6 mg/dL. Of the remaining 18,379 participants 17,922 had values for body weight, height, and appendicular lean soft tissue mass by DXA; and of those 11,240 participants had both serum creatinine and serum cystatin C levels. The sample was treated as a convenience sample and data was obtained from worksheet one of the published data. Serum creatinine and cystatin C levels were corrected as recommended by Selvin et al. (5) and Selvin et al. (6), respectively. Participant characteristics are presented below in **Supplemental Table S1**. **Supplemental Table S2** presents sex-specific skeletal muscle prediction equations, complementing the overall population equations reported in manuscript **Tables 2 and 3**. The script used to extract and preprocess NHANES data is publicly available in the accompanying GitHub repository referenced in the data sharing statement.

Skeletal muscle mass was derived from DXA-measured appendicular lean soft tissue (ALST) mass as reported by McCarthy et al. (2). Serum creatinine alone showed an association with skeletal muscle mass in the full sample, with a cross-validated R² of 0.20 and a root-mean-square error (RMSE) of 5.9 kg (p <0.001; Model A, Table 2). A skeletal muscle mass prediction model using only demographic variables (weight, height, age, and sex) achieved a cross-validated R² of 0.89 and an RMSE of 2.16 kg (Model B), with all demographic variables contributing significantly to the model (p<0.001). Adding serum creatinine to the demographic model resulted in a small improvement in predictive performance, with a cross-validated R² of 0.90 and an RMSE of 2.09 kg (p<0.001; Model C), demonstrating that serum creatinine provides additional, albeit modest, predictive information beyond basic demographic characteristics.

We tested this hypothesis by first examining the association between serum cystatin C and skeletal muscle mass. The correlation between cystatin C and skeletal muscle mass was non-significant (R^2^, 0.0002; RMSE, 6.62 kg; Model D; p = 0.124). By contrast, when cystatin C was added as a covariate (E, **Table 3**), it further increased prediction performance (from Model C: R^2^, 0.90; RMSE, 2.09 kg to Model E: R^2^, 0.91, RMSE, 2.02 kg; p<0.001). Adding waist circumference (WC) to the model resulted in a modest improvement in predictive performance (Model F; R², 0.92; RMSE, 1.89 kg; p<0.001). Sex-specific prediction equations showed lower overall model performance compared with the equations derived from the pooled sex data (**Supplemental Table 1**). WC was included later in this modeling sequence, as it is not consistently available in clinical settings and is prone to measurement error (7).

**Supplemental Table S1**. Sex-specific Skeletal Muscle Prediction Equations Developed Including Serum Creatinine and/or Cystatin C as Model Covariates.

| Model | Equation | R^2^ (RMSE) | P value |
| --- | --- | --- | --- |
|  | Males (N = 5723) |  |  |
| A | 24.46 + 5.04 x SCr | 0.03 (5.11) | <0.001 |
| B | -14.43 - 0.07 x A + 0.16 x Ht + 0.23 x Wt | 0.79 (2.36) | * |
| C | -15.05 - 0.08 x A + 0.14 x Ht + 0.23 x Wt + 4.02 x SCr | 0.81 (2.27) | <0.001 |
| D | 32.64 - 4.14 x SCysC | 0.02 (5.13) | <0.001 |
| E | -13.87 - 0.06 x A + 0.14 x Ht + 0.23 x Wt + 6.15 x SCr - 4.84 x SCysC | 0.83 (2.15) | <0.001 |
| F | 13.2-0.01xA + 0.03xHt + 0.45xWt + 3.86xSCr-3.13xSCysC-0.27xWC | 0.88 (1.83) | <0.001 |
|  | Females (N = 5517) |  |  |
| A | 18.37 + 2.43xSCr | 0.01 (4.27) | <0.001 |
| B | -10.86-0.04xA + 0.12xHt + 0.19xWt | 0.82 (1.8) | * |
| C | -10.91-0.05xA + 0.11xHt + 0.19xWt + 2.58xSCr | 0.83 (1.75) | <0.001 |
| D | 21.04-1.07xSCysC | 0.01 (4.28) | <0.001 |
| E | -9.5-0.04xA + 0.1xHt + 0.2xWt + 4.47xSCr-2.65xSCysC | 0.84 (1.71) | <0.001 |
| F | -4.63-0.03xA + 0.08xHt + 0.24xWt + 4.07xSCr-2.35xSCysC-0.05xWC | 0.85 (1.31) | <0.001 |

*All variables in Model B in both females and males contributed significantly to model performance (p<0.001). The reported p-values correspond to the significance of each variable when it is added to the model.

Abbreviations: A, age (yrs); H, height (cm); RMSE, root-mean square error (kg); SCr, serum creatinine (mg/dL); SCysC, serum cystatin C (mg/ L); Wt, weight (kg); WC, waist circumference (cm).

**Supplementary Data IV. Pennington Biomedical Prediction Model Methods**

The participant data presented in **Supplemental Table S2** was collected from a convenience sample of healthy adults taking part in studies conducted at the New York Obesity Nutrition Research Center’s Body Composition Unit, St Luke-Roosevelt Hospital, New York.  Participants were aged >18 years and BMI >18.5 (kg/m^2^) with the sample characteristics shown in the table below. All participants were ambulatory and weight-stable (<2 kg weight change in previous 6 months) and underwent whole-body MRI scans and fasting blood studies. The methods for evaluating serum leptin, and whole-body MRI for skeletal muscle and adipose tissue are reported in references 8-10, respectively (8-10).

Serum leptin was measured with an enzyme immunoassay and total body adipose tissue mass with whole-body magnetic resonance imaging. Serum leptin levels alone were associated with total body adipose tissue (**Table 4**, Model A; R^2^, 0.36; RMSE 5.84 kg; p< 0.0001). The conventional demographic-alone model (B) predicting total body adipose tissue had an R^2^ and RMSE of 0.77 and 3.5 kg, respectively. Serum leptin added significantly to this model (C), further improving R^2^ (0.81; p< 0.0001) and RMSE (3.19 kg). Lastly, waist circumference added significantly to Model C, again increasing R^2^ (0.82; p< 0.0001) and decreasing RMSE (3.06 kg).

**Supplemental Table S2.** Participant Characteristics.

| **Characteristic** | **Overall**  N = 99^1^ | **male**  N = 51^1^ | **female**  N = 48^1^ |
| --- | --- | --- | --- |
| **Age** (y) | 36 ± 14 | 35 ± 13 | 37 ± 14 |
| **Weight** (kg) | 70 ± 14 | 78 ± 12 | 62 ± 11 |
| **Height** (cm) | 169 ± 10 | 176 ± 6 | 161 ± 7 |
| **Serum Leptin** (ng/mL) | 8 ± 7 | 4 ± 3 | 12 ± 8 |
| **Adipose tissue** (kg) | 20 ± 7 | 18 ± 7 | 21 ± 8 |
| **Waist circumference** (cm) | 81 ± 11 | 85 ± 9 | 76 ± 10 |
| ^1^ Results are Mean ± SD. | | | |

**Supplemental References**

1. Pasiakos SM, Berryman CE, Karl JP, Lieberman HR, Orr JS, Margolis LM et al. Physiological and psychological effects of testosterone during severe energy deficit and recovery: A study protocol for a randomized, placebo-controlled trial for Optimizing Performance for Soldiers (OPS). *Contemp Clin Trials*. 2017 Jul;58:47-57.

2. McCarthy C, Schoeller D, Brown JC, Gonzalez MC, Varanoske AN, Cataldi D et al. D(3) -creatine dilution for skeletal muscle mass measurement: historical development and current status. *J Cachexia Sarcopenia Muscle*. 2022 Dec;13(6):2595-607.

3. Pierson RN, Jr., Wang J, Heymsfield SB, Russell-Aulet M, Mazariegos M, Tierney M et al. Measuring body fat: calibrating the rulers. Intermethod comparisons in 389 normal Caucasian subjects. *Am J Physiol*. 1991 Jul;261(1 Pt 1):E103-8.

4. Wang ZM, Sun YG, Heymsfield SB. Urinary creatinine-skeletal muscle mass method: a prediction equation based on computerized axial tomography. *Biomed Environ Sci*. 1996 Sep;9(2-3):185-90.

5. Selvin E, Manzi J, Stevens LA, Van Lente F, Lacher DA, Levey AS et al. Calibration of serum creatinine in the National Health and Nutrition Examination Surveys (NHANES) 1988-1994, 1999-2004. *Am J Kidney Dis*. 2007 Dec;50(6):918-26.

6. Selvin E, Juraschek SP, Eckfeldt J, Levey AS, Inker LA, Coresh J. Calibration of cystatin C in the National Health and Nutrition Examination Surveys (NHANES). *Am J Kidney Dis*. 2013 Feb;61(2):353-4.

7. Mason C, Katzmarzyk PT. Variability in waist circumference measurements according to anatomic measurement site. *Obesity (Silver Spring)*. 2009 Sep;17(9):1789-95.

8. Hwaung P, Bosy-Westphal A, Muller MJ, Geisler C, Heo M, Thomas DM et al. Obesity Tissue: Composition, Energy Expenditure, and Energy Content in Adult Humans. *Obesity (Silver Spring)*. 2019 Sep;27(9):1472-81.

9. Kim J, Heshka S, Gallagher D, Kotler DP, Mayer L, Albu J et al. Intermuscular adipose tissue-free skeletal muscle mass: estimation by dual-energy X-ray absorptiometry in adults. *J Appl Physiol (1985)*. 2004 Aug;97(2):655-60.

10. Wang Z, Zhu S, Wang J, Pierson RN, Jr., Heymsfield SB. Whole-body skeletal muscle mass: development and validation of total-body potassium prediction models. *Am J Clin Nutr*. 2003 Jan;77(1):76-82.
